# Lead induced structural and functional damage and microbiota dysbiosis in the intestine of crucian carp (*Carassius auratus*)

**DOI:** 10.3389/fmicb.2023.1239323

**Published:** 2023-09-04

**Authors:** Haisu Liu, Hang Zhang, Qianxun Yu, Sanshan Zhang, Xiao Tu, Fenghong Zhuang, Shengli Fu

**Affiliations:** ^1^Research Center of Harmful Algae and Marine Biology, Key Laboratory of Eutrophication and Red Tide Prevention of Guangdong Higher Education Institutes, College of Life Science and Technology, Jinan University, Guangzhou, China; ^2^School of Life Sciences, South China Normal University, Guangzhou, China; ^3^Hubei Water Resources Research Institute, Hubei Water Resources and Hydropower Science and Technology Information Center, Wuhan, China; ^4^Hubei Institute of Product Quality Supervision and Inspection, Wuhan, China

**Keywords:** Pb, *Carassius auratus*, oxidative stress, immune response, intestinal microbiota

## Abstract

Lead (Pb) is a hazardous pollutant in water environments that can cause significant damage to aquatic animals and humans. In this study, crucian carp (*Carassius auratus*) were exposed to waterborne Pb for 96 h; then, histopathological analysis, quantitative qPCR analysis, and 16S high-throughput sequencing were performed to explore the effects of Pb on intestinal bioaccumulation, structural damage, oxidative stress, immune response, and microbiota imbalance of *C. auratus*. After Pb exposure, the intestinal morphology was obviously damaged, including significantly increasing the thickness of the intestinal wall and the number of goblet cells and reducing the depth of intestinal crypts. Pb exposure reduced the mRNA expressions of *Claudin-7* and *villin-1* while significantly elevated the level of *GST, GSH, CAT, IL-8, IL-10, IL-1*, and *TNF-*α. Furthermore, 16S rRNA analysis showed that the Shannon and Simpson indices decreased at 48 h after Pb exposure, and the abundance of pathogenic bacteria (Erysipelotrichaceae, Weeksellaceae, and Vibrionaceae) increased after Pb exposure. In addition, the correlation network analysis found that *Proteobacteria* were negatively correlated with *Firmicutes* and positively correlated with *Bacteroidetes*. Functional prediction analysis of bacteria speculated that the change in intestinal microbiota led to the PPAR signaling pathway and peroxisome function of the intestine of crucian carp was increased, while the immune system and membrane transport function were decreased. Finally, canonical correlation analysis (CCA) found that there were correlations between the intestinal microbiota, morphology, antioxidant factors, and immune factors of crucian carp after Pb exposure. Taken together, our results demonstrated that intestinal flora dysbiosis, morphological disruption, oxidative stress, and immune injury are involved in the toxic damage of Pb exposure to the intestinal structure and function of crucian carp. Meanwhile, Pb exposure rapidly increased the abundance of pathogenic bacteria, leading to intestinal disorders, further aggravating the damage of Pb to intestinal structure and function. These findings provide us a basis for the link between gut microbiome changes and heavy metal toxicity, and gut microbiota can be used as biomarkers for the evaluation of heavy metal pollution in future.

## Graphical Abstract

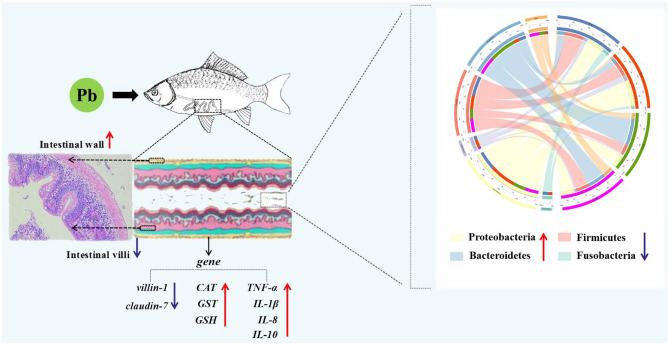

## 1. Introduction

With the development of the economy and the increase in population, the problem of environmental pollution is becoming more and more serious (Yu Y. Y. et al., 2021). Aquatic system, as an important part of the biosphere, has been seriously threatened by heavy metal pollution in water in the past few decades and has become a worldwide environmental problem (Shi et al., 2020; Yin et al., 2020; Rout et al., 2022a; Chen et al., 2023). In the past 5 years, the accumulation of heavy metals has been found in many water areas of China, such as Yangtze, Yellow, and Pearl Rivers (Xie et al., 2022; Chen et al., 2023; Liang et al., 2023). Heavy metal pollutants in the water environment were not only toxic to aquatic products but may also be absorbed by fish and passed up the food chain to humans through bioaccumulation (Francesco et al., 2014; Mario et al., 2019; Liu et al., 2020). As the major toxic heavy metals in the environment, Lead (Pb) is a key pollutant in environmental monitoring and contamination control, which has high toxicity to humans and organisms in the environment and has extensive pollution in the atmosphere, soil, and water (Chen et al., 2016; Behera et al., 2020, 2023; Shi et al., 2020; Parida et al., 2022). In 2015, Qi et al. found that the Pb content in the Fen River reached 21.2 mg/kg, and Huang et al. found that the Pb content in river soil caused by pollution in the Yunnan mining area reached 3,030.0 mg/kg in 2016 (Qi et al., 2015; Huang et al., 2016). Pb exposure has great toxic effects on the physiological, behavioral, and biochemical functions of animals. Previous studies have shown that Pb causes the accumulation of heavy metals, histopathology, and neurotoxicity in fish (Lee et al., 2019; Shahjahan et al., 2022). Lead accumulation can also cause oxidative stress in aquatic organisms and damage the antioxidant function of the body (Guo et al., 2021; Kumar et al., 2021). Therefore, several antioxidant markers, such as antioxidant enzymes (GST and CAT) and glutathione (GSH), are often used to evaluate the toxic effects of Pb-induced oxidative stress. However, with the development of toxicological research, the continued development of more reliable biomarkers to provide a more comprehensive monitoring and evaluation of pollution levels is warranted.

Intestinal microbiota is an intricate and unique ecosystem that is symbiotic with the host and is closely related to the host's antioxidant and immunity, as well as survival, metabolism, and behavior (Premysl et al., 2011; Daga et al., 2013; Wang et al., 2019; Meijerink et al., 2020; Zhang et al., 2020). Abnormal intestinal flora can affect host physiological activities, such as metabolic, inflammatory, and immune diseases, even increase host susceptibility to pathogens (Qi et al., 2019; Liu et al., 2020; Yu Z. W. et al., 2021). Stable intestinal microbiota structure is of great help to maintain the growth and development of the intestine of fish. On the other hand, foreign substances can interfere with the normal growth of the gut microbiota structure. For example, external low-temperature environment can improve the relative abundance of vibrio in the intestine, reduce the relative abundance of other probiotics, and change the structure of intestinal microbiota, leading to the occurrence of fish diseases (LeaMaster et al., 1997). Because the intestinal microbiota is a sensitive plastic factor responding to environmental changes, intestinal microbes have been used as a new biomarker for toxicity assessment in mammals (Candela et al., 2012; Li et al., 2021). Moreover, in recent years, the correlation between intestinal microbiota and heavy metal pollution has attracted attention (Duan et al., 2021). Therefore, understanding the toxic effect of waterborne Pb on fish intestinal microbiota, researching the correlation between microbes and the structure and function of the intestine under Pb pollution, has important research value for the development of fish intestinal microbiota as a biomarker for the assessment of aquatic environment pollution.

Fish, as a high tropic level in aquatic ecosystems, are widely present in aquatic environments around the world (Oost et al., 2003; Wang et al., 2021; Carlos et al., 2022; Kristine et al., 2022; Souza et al., 2022). Because of the accumulation of large amount of water pollutants through the food chain and bioaccumulation, fish are often considered as the suitable bioindicators of contaminated aquatic ecosystem (Yin et al., 2020; Mario et al., 2022). Crucian carp, belonging to the carp family, omnivorous, is one of the most common fish in freshwater environments (Dong and Li, 1994; Radke and Kahl, 2002; Li T. et al., 2015). Crucian carp is widely distributed in Eurasia, with excellent growth characteristics, good taste, and wide applicability in aquaculture systems and is an important commercial aquaculture fish in the world, and the annual global aquaculture production is millions of tons (FAO; Food and Agriculture Organization of the United Nations) (Rhee et al., 2014; Liu et al., 2017). As an energy carrier from lower to higher nutrient levels, the Pb accumulation of crucian carp would raise the risk of fish consumption (Oost et al., 2003). Thus, it is necessary to explore the toxicity of Pb to crucian carp to evaluate its hazards to aquatic organisms. In this study, crucian carp were exposed to simulated ponds with added lead contamination sources. We used inductively coupled plasma mass spectrometry (ICP-MS), histopathological symptom observation, and quantitative qPCR analysis to investigate the bioaccumulation levels of lead in the fish intestine, intestinal structural damage, and the expression levels of immune and antioxidant factors. In addition, the dynamics of the fish intestinal microbiota were detected by using 16s high-throughput sequencing technology to provide a detailed analysis report on the response of crucian carp to Pb stress. This will help to further understand the relationship between the intestinal structure, function, and microbial community of crucian carp under lead pollution and provide theoretical reference and experimental basis for intestinal microbes as a new water pollution toxicity biomarker.

## 2. Materials and methods

### 2.1. Fish maintenance and Pb exposure treatment

In this study, juvenile crucian carp (weight 13.4 ± 0.5 g, length 10.5 ± 0.3 cm) were provided by an aquaculture farm in Huadu, Guangzhou, China. The experiment was carried out at the aquaculture base of South China Normal University. Before the Pb exposure experiment, acclimation was performed as usual for 2 weeks (Liu et al., 2021). During the domestication period, the death rate of the experimental fish was <2% and had no clinical signs of disease.

Acute toxicity test and half lethal concentration (LC_50_) test for 96 h of Pb(NO_3_)_2_ were carried out according to the previous method (Pirsaheb et al., 2019). The 96-h LC_50_ of Pb for crucian carp was 216.62 mg/L. The safe concentration (SC) was calculated by multiplying the LC_50_ values with an application factor of 0.1 (Liu et al., 2020).

Sixty healthy crucian carp were randomly selected and cultured in three 1 m^3^ aquariums, 20 in each aquarium. Then, Pb(NO_3_)_2_ was added to make the Pb concentration in the culture water reach SC (21.66 mg/L) and maintained during the experiment. The posterior intestine of a fish was taken from each aquarium at 0 h (J 0h), 6 h (J 6h), 48 h (J 48h), and 96 h (J 96h), after Pb(NO_3_)_2_ exposure (*n* = 3). Fish were anesthetized with 0.05% tricaine methane sulfonate (MS-222; Aladdin, China) before sampling. A part of the posterior intestine of each fish was stored in Bouin's solution (Servicebio, China), and the other part of the posterior intestine of each fish was stored at −80°C after quick freezing in liquid nitrogen.

### 2.2. Pb content detection

The intestine from four groups (each group, *n* = 3) were collected, homogenized, and moved to the microwave digestion apparatus (Qiyao, China) for digestion (Shirin et al., 2015). Pb standard solution (Merck, Darmstadt, Germany) was injected into the inductively coupled plasma mass spectrometry (ICP-MS; Agilent, USA), to make the standard curve. Then, samples after microwave digestion were injected into the ICP-MS. According to the standard curve, the Pb content in the posterior intestine was obtained by referring to the previous methods (Li et al., 2020; Liu et al., 2020).

### 2.3. Intestinal histopathological examination

The intestine of crucian carp was fixed with Bouin's solution, embedded in paraffin, sectioned at 4 μm thickness, and stained with hematoxylin and eosin (HE). Finally, the intestinal tissue was observed under a polarized light microscope (Leica, Germany) (Tan et al., 2018).

### 2.4. qRT-PCR analysis

The total RNA from all samples was extracted by using TRIzol reagent (Vazyme, China), according to the manufacturer's instructions (Fu et al., 2019), and its quality and concentration were detected by electrophoresis in 1% agarose gel and NanoDrop 2000 spectrophotometer (NanoDrop Technologies, USA) (Fu et al., 2018). Then, using PrimeScript RT Reagent Kit (Takara, China) to synthesize cDNA and using the real-time PCR system (Bio-Rad, USA) to perform qRT-PCR (Huang et al., 2018). β-actin gene of crucian carp (GenBank accession NO. AB039726.2) was used as an internal control for the normalization of gene expression. Primers of genes (β*-actin, Claudin-7, villin-1, GST, GSH, IL-1, IL-8, IL-10*, and *TNF-*α) for qRT-PCR were designed by using Primer Premier 5 (Premier, Canada). The primer sequences are presented in Table 1.

**Table 1:** The primer sequences used in this study for qRT-PCR.

**Primers**	**Nucleotide sequence (5^′^ → 3^′^)**
RT-*β-actin*-F	GGAAATCGCTGCCCTGGT
RT-*β-actin*-R	ATGCCAACCATCACTCCCTG
RT-*IL-8*-F	CATTTTCCTTGGATTCCTGACC
RT-*IL-8*-R	GTGTGAGTTTGGAGGGAAGAGC
RT-*TNF-α*-F	TGTGGGGTCCTGCTGGCT
RT-*TNF-α*-R	GCCTGGTCCTGGTTCTGTTTC
RT-*IL-10*-F	TTGCTCATTTGTGGAGGGCT
RT-*IL-10*-R	AAGGACTGTTGATGTGCTGTTGC
RT-*CAT*-F	CCATCTCCAACGGCAACTTC
RT-*CAT*-R	TCAAACGGATTCCACTGCCA
RT-*GST*-F	CCTGAACTACATCGCTGGAAAAT
RT-*GST*-R	CGGTTTGTTTTCAGGCGGT
RT-*GSH*-F	TTGCCAAGTCCTACAATGCGG
RT-*GSH*-R	TTCCTTCAGCCACTTCCACAGA
RT-*villin-1*-F	CTTGTGCCCTGTCCACCTAATA
RT-*villin-1*-R	GCCTTGCCCAGCCAATAAT
RT-*Claudin-7*-F	CAAGGTGTACGACTCCATCCTACA
RT-*Claudin-7*-R	CACTTCATGCCCATGCTGG

### 2.5. DNA extraction and 16s rRNA gene amplification sequence analysis

All samples were sent to Guangzhou JiRui Gene Technology Co. Ltd. (China) for extraction of DNA and PCR amplification by the Illumina MiSeq Sequencing platform. PCR was performed from V3–V4 variable regions of 16S rRNA to taxonomically identify and analyze the bacteria (Liu et al., 2020; Dixit et al., 2021).

### 2.6. Statistical analysis

Unless otherwise stated, all experiments were performed with triplicate samples (*n* = 3), and the data are presented as means ± standard deviation. Before statistical analysis, the data tested for normality (Shapiro–Wilk) and homogeneity of variance (Levene's test) were assessed, and the assumptions were met. Differences in the expression levels of genes related to intestinal structure and function, intestinal structure-related indicators (intestinal wall thickness, crypt depth, and goblet cell number), relative abundance of intestinal microbiota, and functional proportion of intestinal microbiota were obtained by one-way ANOVA test. Graphs of Pb concentration and qPCR analysis were generated using GraphPad Prism 9, a number of intestinal goblet cells, intestinal wall thickness, and crypt depth were calculated using Image J, and the results were presented as means ± standard deviation (*n* = 3). Intestinal microbial operational taxonomic unit (OUT) classification statistics were performed using UPARSE. Alpha diversity analysis was performed using Mothur 1.30.2. Microbial functional prediction analysis was performed using the Kyoto Encyclopedia of Genes and Genomes database (KEGG, http://eggnog.embl.de/). Venn diagram analysis, network relationship analysis, and canonical correlation analysis (CCA) of microbiota were performed using the R software package (VennDiagram, Hmisc, and Vegan package). Statistical significance was set at *P* < 0.05 (^*^), and very significance was set at *P* < 0.01 (^**^) for all tests.

## 3. Results

### 3.1. Pb accumulation and histological analysis

**Figure 1:**
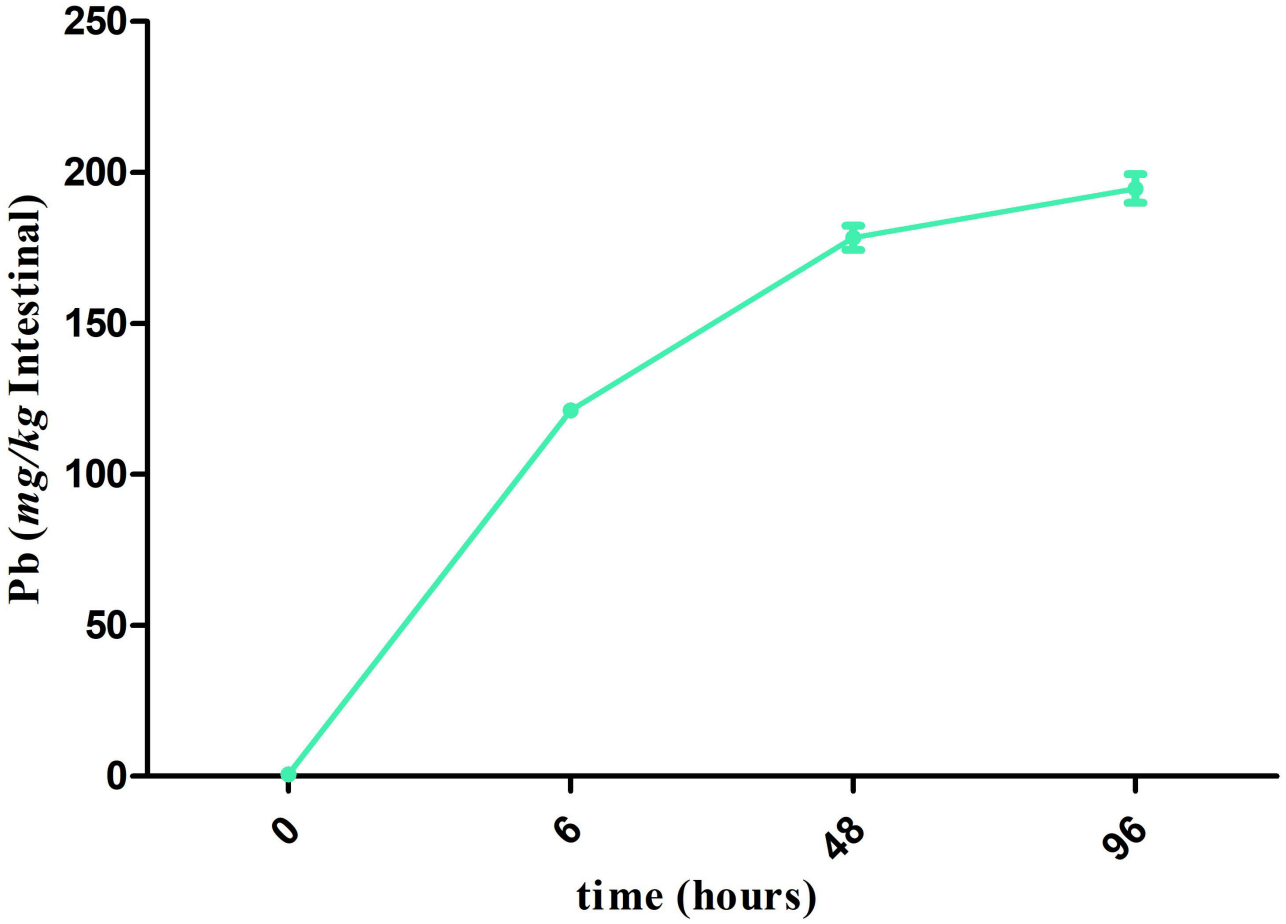
Accumulation of Pb in the crucian carp after Pb exposure during 96 h. The points in the line graphs represent the means ± SD of Pb content in the intestine of crucian carp (*n* =3).

Compared with the J 0 h group, the accumulation of Pb in the intestine was significantly increased (*P* < 0.05) in all the other groups, and the accumulation level of Pb was the highest (194.68 mg/kg) in the J 96 h group (Figure 1).

**Figure 2:**
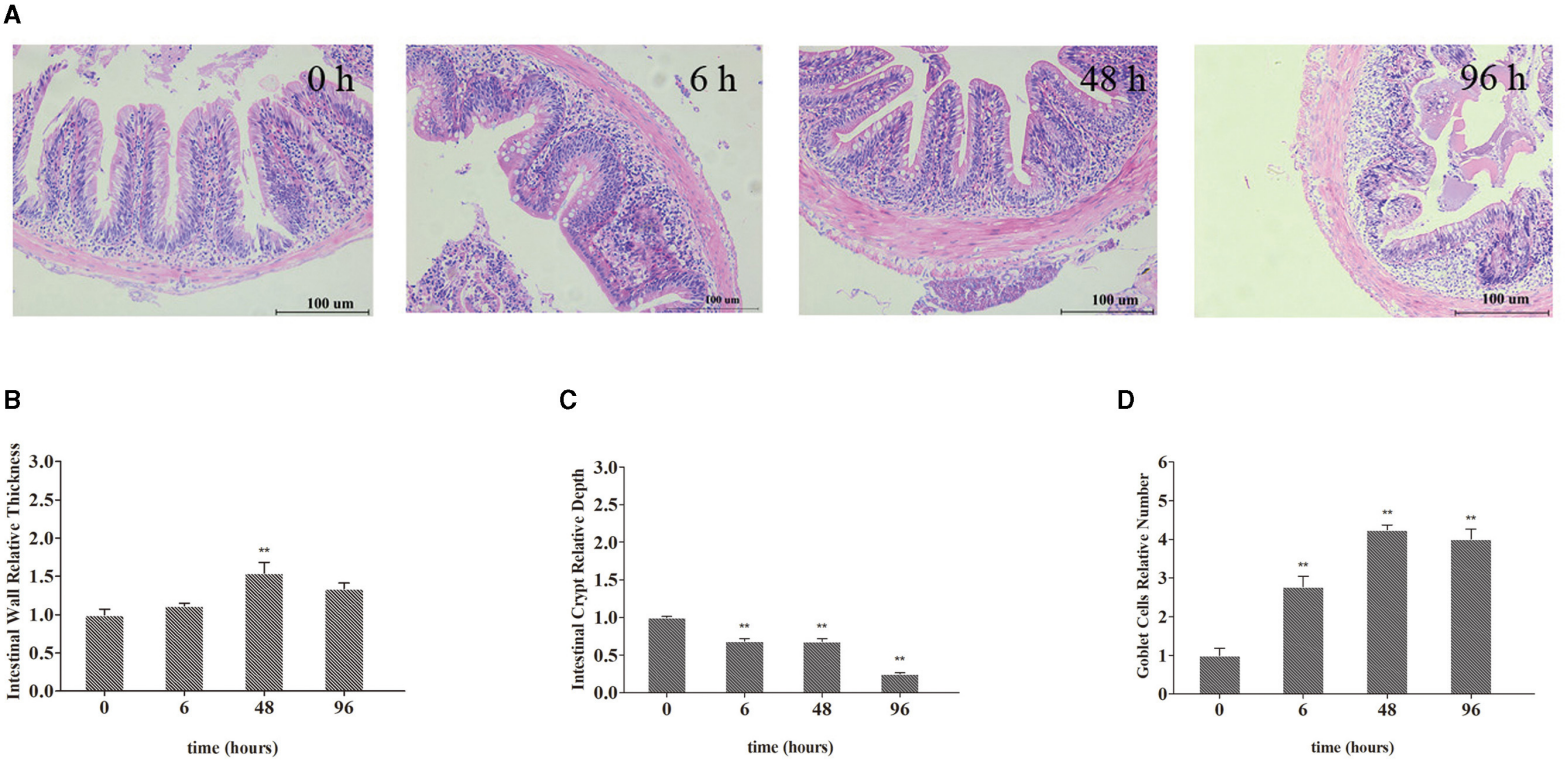
Representative micrographs of intestines from crucian carp. **(A)** Intestinal wall relative thickness. **(B)** Intestine crypt relative depth. **(C)** Goblet cells' relative number **(D)**. Leukocyte infiltration occurs in the area indicated by the arrow. The bar plot denotes means ± SD of three replications, and the asterisk represents a statistically significant difference (*0.01 < *P* < 0.05 and **P < 0.01).

The intestinal morphological changes are presented in Figure 2A. Compared with the J 0 h group, intestinal wall thickening, intestinal villi shortening, goblet cells increasing, and slight phagocytosis of leukocytes were observed in the 6, 48, and 96 h groups (Figures 2B–D). Specifically, after treated with Pb, the relative intestinal wall thickness was increased significantly and reached the highest level at 48 h (up to 1.54-fold, *P* < 0.01). After Pb exposure, the relative depth of intestinal crypts decreased and reached the lowest level at 96 h (down to 0.25-fold, *P* < 0.01) after Pb exposure, while the goblet cells' number of intestine increased and reached the highest level at 48 h (up to 4.24-fold, *P* < 0.01).

### 3.2. The expression of structure, antioxidant, and immune factor-related genes in the intestines

**Figure 3:**
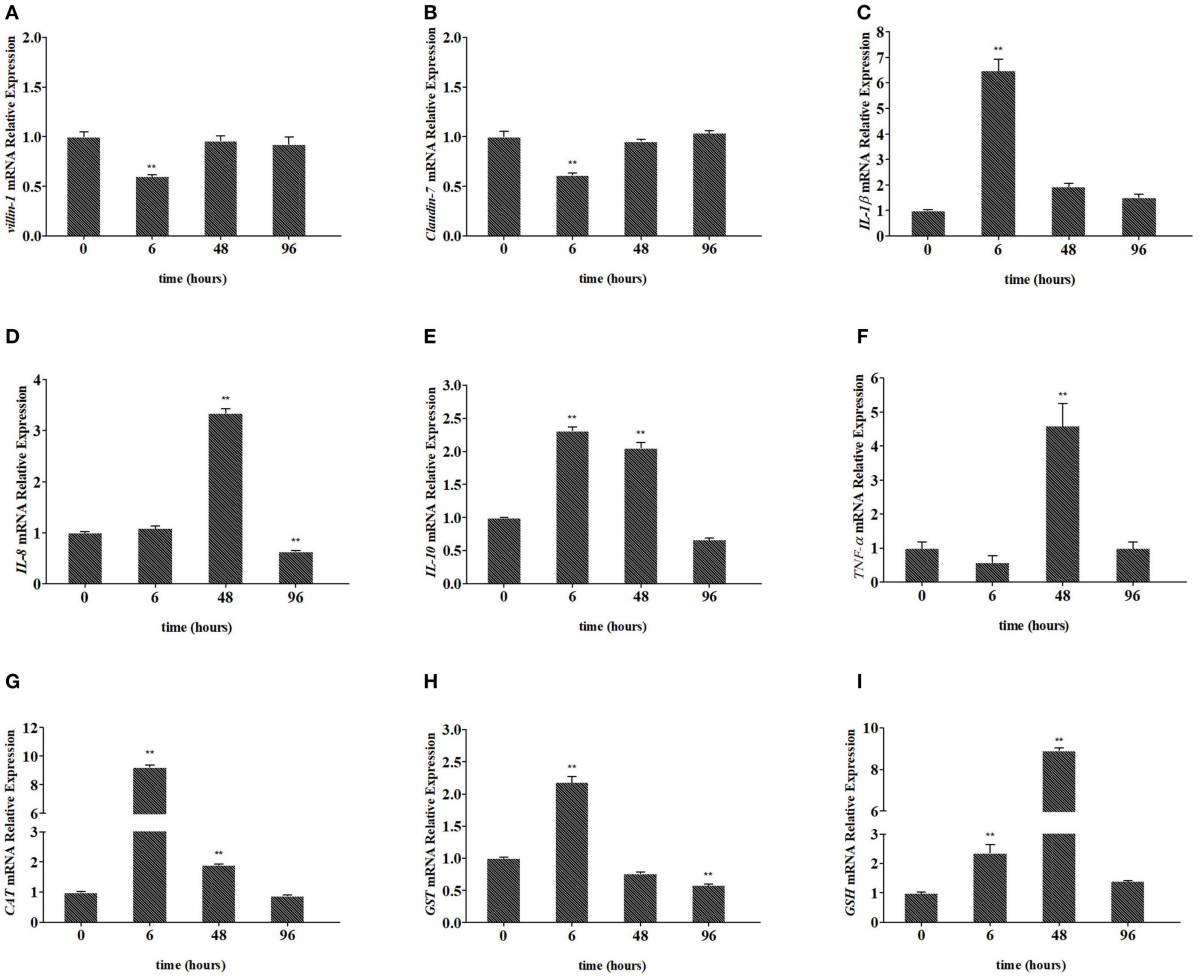
Relative expression of *villin-1* **(A)**, *Claudin-7* **(B)**, *IL-1*β **(C)**, *IL-8* **(D)**, *IL-10* **(E)**, *TNF-*α **(F)**, *CAT* **(G)**, *GST* **(H)**, and *GSH* **(I)** in the intestines of crucian carp. The mRNA levels of each gene were normalized to that of β-actin. The bar plot denotes means ± SD of three replications, and the asterisk represents a statistically significant difference (*0.01 < *P* < 0.05 and **P < 0.01).

As shown in Figure 3, the expression of *villin-1* and *Claudin-7* in the intestine decreased significantly and reached the lowest level at 6 h (down to 0.60-fold, *P* < 0.01, and 0.62-fold, *P* < 0.01, respectively; Figures 3A, B) and returned to the normal level at 96 h, after Pb exposure. In Figures 3C, E, the expression of *IL-1*β and *IL-10* in the intestine significantly increased to the highest level at 6 h (up to 6.48-fold, *P* < 0.01, and 2.31-fold, *P* < 0.01, respectively), after Pb exposure and gradually recovered later. In Figures 3D, F, the expression of *IL-8* and *TNF-*α in the intestine significantly increased to the highest level at 48 h (up to 3.34-fold, *P* < 0.01, and 4.59-fold, *P* < 0.01, respectively) and gradually recovered at 96 h. As shown in Figures 3G, H, the expression of *CAT* and *GST* in the intestine significantly increased to the highest level at 6 h (up to 9.24-fold, *p* < 0.01, 2.18-fold, *p* < 0.01, respectively), after Pb exposure and gradually recovered later. In Figure 3I, the expression of *GSH* in the intestine significantly increased to the highest level at 48 h (up to 8.91-fold, *P* < 0.01) and gradually recovered at 96 h.

### 3.3. Microbiota signature of the intestine of crucian carp

A total of 792,426 clean reads were obtained from the four groups, with valid sequences ranging from 21,391 to 152,300. Cluster analysis of the crucian carp's intestinal microbiota was divided into 592 OTUs, excluding monad sequences. There were 389 OTUs in the J 0 h group, 327 OTUs in the J 6 h group, 284 OTUs in the J 48 h group, and 453 OTUs in the J 96 h group, and 307 OTUs coexisted in the four groups (Supplementary Figure S1). The rarefaction curves of each sample tend to be saturated (Supplementary Figure S2). The good coverage (more than 99%) of sequencing and alpha diversity of microbiota are shown in Table 2. The trend of ACE and Chao1 was increased at 96 h (Supplementary Figures S3A, B), while Shannon and Simpson indices were decreased at 48 h after Pb exposure (Supplementary Figures S3C, D).

**Table 2:** Alpha diversity (diversity and richness) indexes as calculated by MOTHUR software (ver. 1.30.0).

**Samples**	**Total sequences passed quality check**	**Total OTUs**	**Alpha diversity indexes** ^**a**^				**Good's coverage**
			**Ace index**	**Chao1 index**	**Simpson index**	**Shannon index**	
J 0 h	213,701	725	177.32 ± 78.55	183.07 ± 76.65	0.89 ± 0.03	4.63 ± 0.84	0.999
J 6 h	195,575	731	310.09 ± 56.07	315.60 ± 56.15	0.84 ± 0.08	4.34 ± 0.56	0.998
J 48 h	266,889	657	337.33 ± 11.43	349.08 ± 20.11	0.79 ± 0.06	3.73 ± 0.54	0.998
J 96 h	116,261	983	274.15 ± 74.85	267.67 ± 80.42	0.94 ± 0.02	5.48 ± 0.66	0.997

Operational taxonomic units (OTUs) are defined at 97% sequence similarity.

^a^Values represent the average ± SD (each mean value of three determinations).

### 3.4. Composition analysis of intestinal microbiota

**Figure 4:**
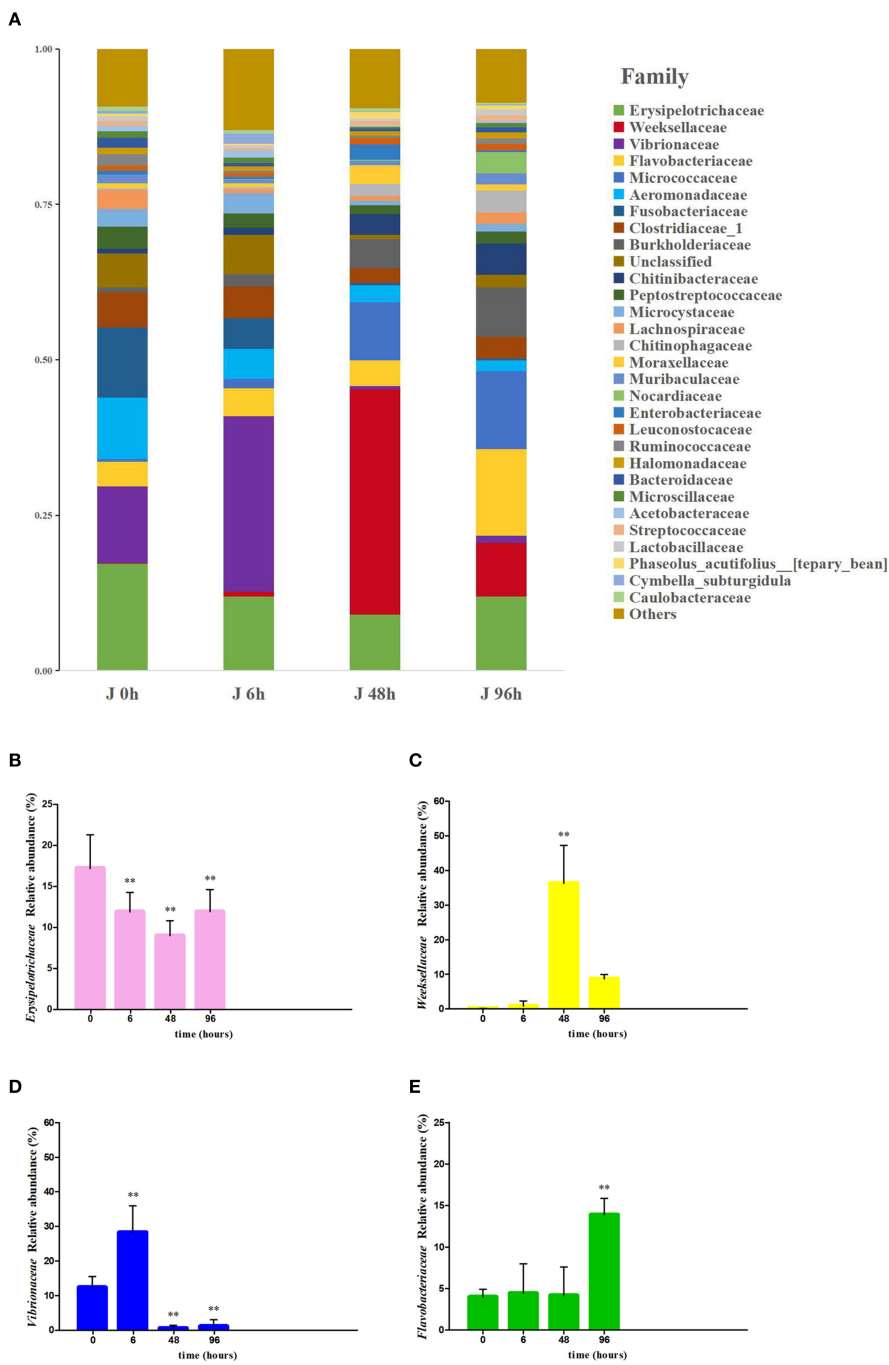
Effects of Pb exposure on the composition and relative abundance of microbiota crucian carp's intestines at the family level **(A)**. The relative abundance of Erysipelotrichaceae **(B)**, Weeksellaceae **(C)**, Vibrionaceae **(D)**, and Flavobacteriaceae **(E)** was measured. The asterisk represents a statistically significant difference (*0.01 < *P* < 0.05 and **P < 0.01).

At the phylum level, 19 bacterial phyla were identified in the intestine of crucian carp, and the relative abundance of (More than 5% in at least one sample) *Proteobacteria, Cyanobacteria, Firmicutes*, and *Bacteroidetes* were 22.80–45.95%, 2.46–10.82%, 16.56–34.08%, and 10.05–47.78%, respectively. The relative abundance of *Firmicutes* (34.08%), *Proteobacteria* (45.95%), *Bacteroidete* (47.78%), and *Bacteroidete* (31.77%) were the highest in the J 0 h group, J 6 h group, J 48 h group, and J 96 h group, respectively (Supplementary Figure S4). At the family level, 29 bacteria were classified by the community barplot analysis (Figure 4A), among which four main bacteria were selected for further analysis and they were Erysipelotrichaceae, Weeksellaceae, Vibrionaceae, and Flavobacteriaceae. The relative abundance of Erysipelotrichaceae was significantly decreased to the lowest level in the J 48 h group (down to 0.52-fold, *P* < 0.01; Figure 4B), while the abundance of Vibrionaceae, Weeksellaceae, and Flavobacteriaceae was significantly increased to the highest level in the J 6 h group (up to 2.29-fold, *P* < 0.01), J 48 h group (up to 353.88-fold, *P* < 0.01), and J 96 h group (up to 3.50-fold, *P* < 0.01), respectively (Figures 4C–E).

### 3.5. Analysis of significantly different intestinal microbiota deviation values

**Figure 5:**
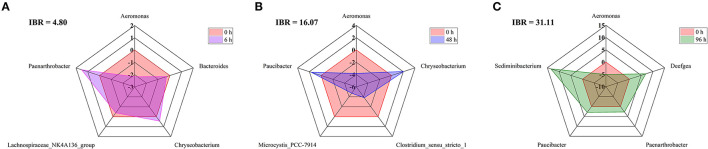
IBR index, Biomarker bias index, and associated star plots of the main difference bacteria at the genus level between 0 h, 6 h **(A)**, 48 h **(B)**, and 96 h **(C)** in the intestine of crucian carp.

Metastats analysis was used to screen the bacteria with significant differences in abundance among each group of crucial carp intestine, and the Integrated Biomarker Response (IBR) index and deviation index of the main difference bacteria at 0 h, 6 h, 48 h, and 96 h were calculated (Figure 5). Compared with 0 h, the IBR indexes for 6 h, 48 h, and 96 h were 4.80, 16.07, and 31.11, respectively; the deviation values of *Aeromonas, Bacteroides*, and *Lachnospiraceae_NK4A136_group* were decreased at 6 h, the deviation values of *Aeromonas, Clostridium_sensu_stricto_1*, and *Microcystis_PCC-7914* were decreased at 48 h, and the deviation values of *Aeromonas* were decreased at 96 h. Instead, the deviation values of *Chryseobacterium* and *Paenarthrobacter* were increased at 6 h. The deviation values of *Chryseobacterium* and *Paucibacter* were increased at 48 h, and the deviation values of *Deefgea, Paenarthrobacter, Paucibacter*, and *Sediminibacterium* were increased at 96 h.

### 3.6. Intestinal microbiota network analysis

**Figure 6:**
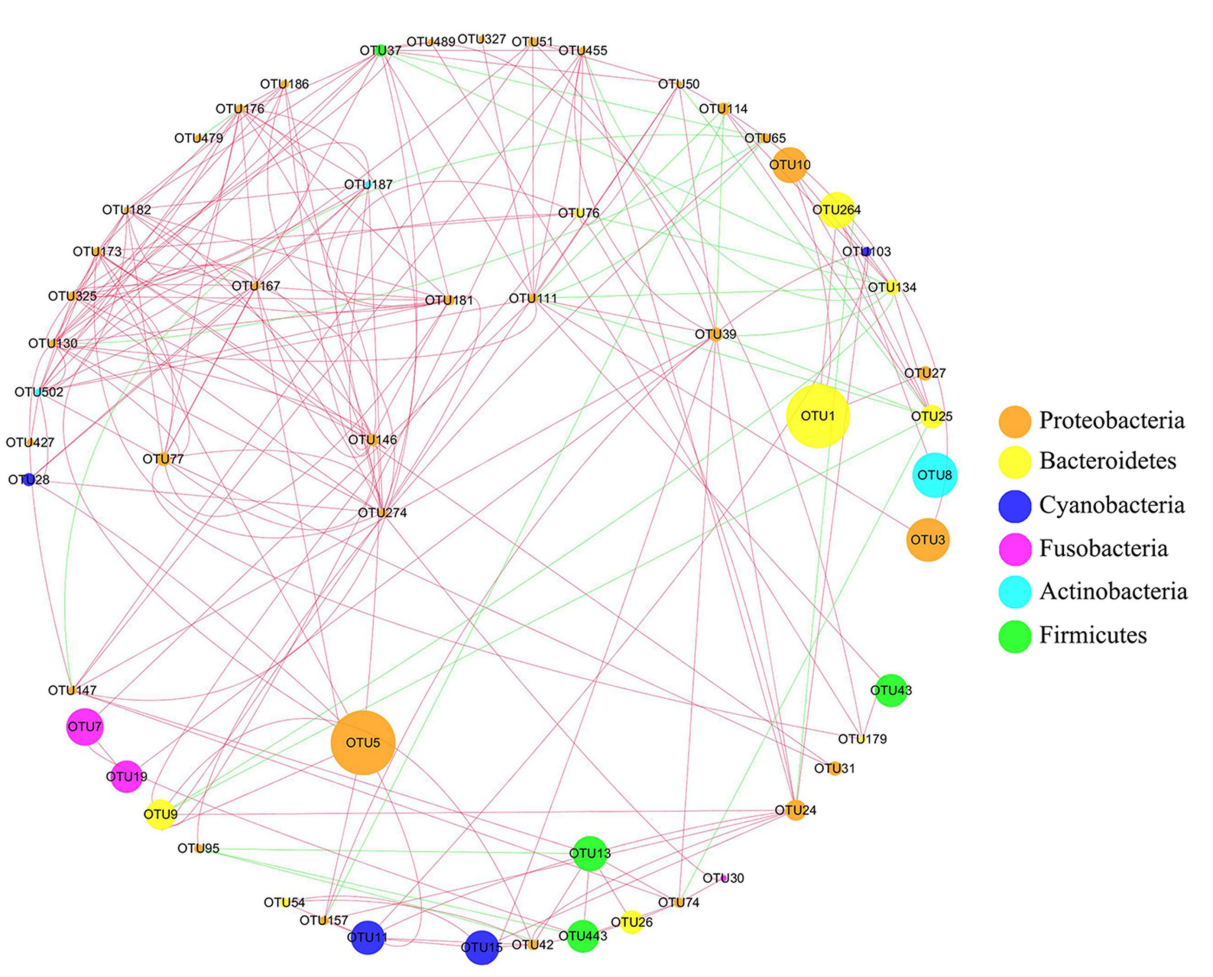
Network analysis of intestinal microbiota in crucian carp after Pb exposure. The circular nodes represent the species taxonomy of OTUs, and the size represents the abundance of OTUs. The lines indicate correlation, red indicates positive correlation, and green indicates negative correlation.

According to the correlation network analysis of the abundance of OTUs, most OTUs of *Proteobacteria* were negatively correlated with *Firmicutes*, while most OTUs of *Proteobacteria* were positively correlated with *Bacteroidetes* (Figure 6). The most abundant taxa were OTU5 and OTU1 (*Chryseobacterium soldanellicola*). Among them, OTU1 (belonging to Weeksellaceae) was positively correlated with OTU10 (belonging to Chitinibacteraceae), OTU27 (belonging to Chitinibacteraceae), and OTU264 (belonging to Flavobacteriaceae). Meanwhile, OTU5 (belonging to Vibrionaceae) was positively correlated with OTU9 (belonging to Flavobacteriaceae), OTU157 (belonging to Burkholderiaceae), and OTU146 (belonging to Burkholderiaceae).

### 3.7. PICRUST analysis of intestinal microbiota in crucian carp

**Figure 7:**
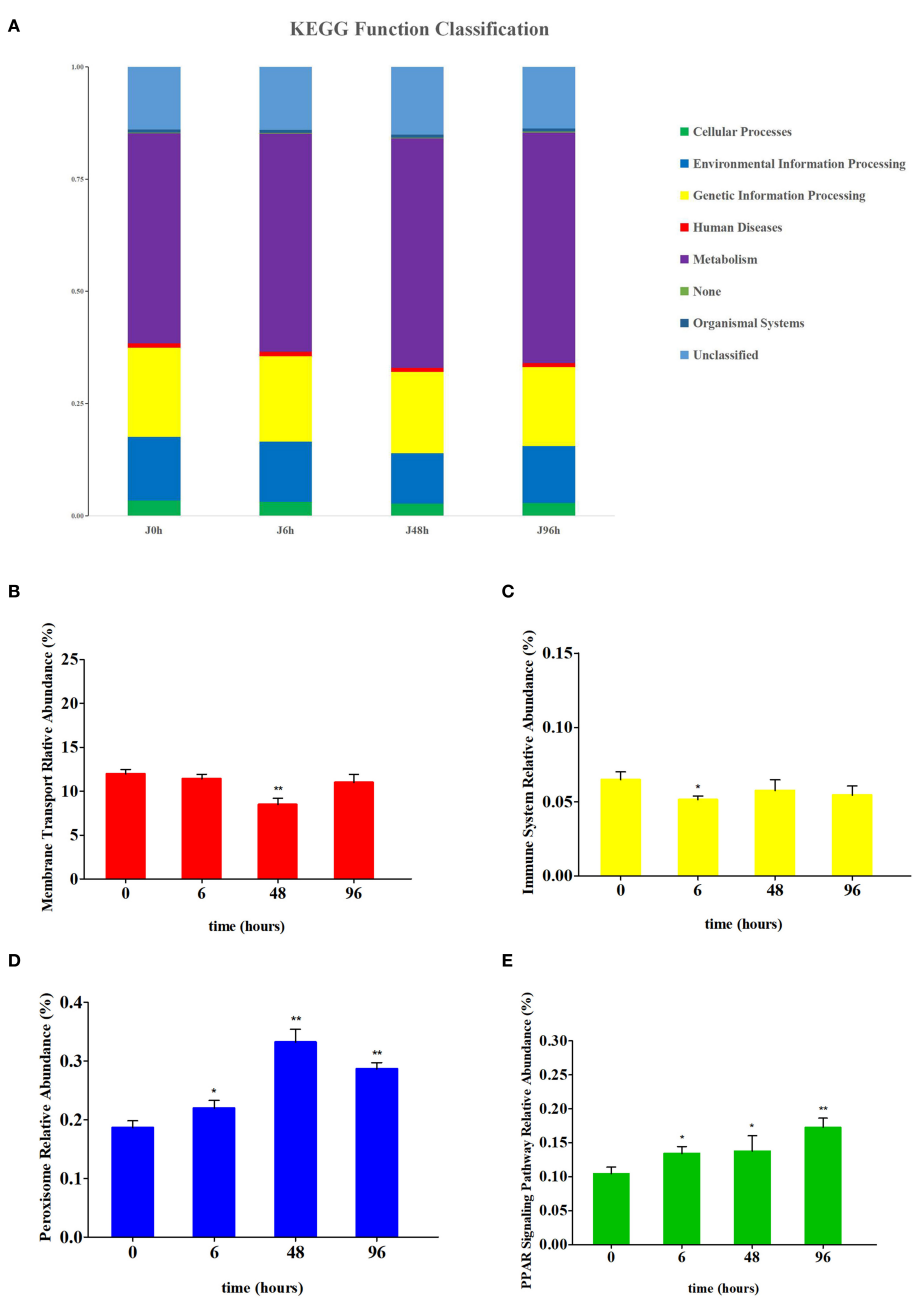
Comparison of the relative abundance of PICRUSt-generated function profile of crucian carp gut in each group. **(A)** The KEGG function classification at level 1 of each group. **(B)** Relative abundance changes of membrane transport in the intestines of crucian carp. **(C)** Relative abundance changes of the immune system in the intestines of crucian carp. **(D)** Relative abundance changes of peroxisome in the intestines of crucian carp. **(E)** Relative abundance changes of PPAR signaling pathway in the intestines of crucian carp. Asterisk represents a statistically significant difference (*0.01 < *P* < 0.05 and **P < 0.01).

Based on the pathway comparison analysis of Kyoto Encyclopedia of Genes and Genomes (KEGG), the influence of intestinal microbiota change on the intestinal function of crucian carp were predicted. Level 1 functions were divided into six categories, among which the metabolism was the most abundant (Figure 7A). At the level 2, membrane transport and the immune system function were significantly changed after Pb exposure (Supplementary Table S1). Similarly, the peroxisome and PPAR signaling pathway function were significantly changed at level 3 (Supplementary Table S2). Functional changes in membrane transport, immune system, peroxisome, and PPAR signaling pathways are shown in Figures 7B–E, respectively. After Pb exposure, the relative abundance of peroxisome and PPAR signaling pathway functions in the intestine increased significantly, while membrane transport and immune system decreased significantly. The relative abundance levels of membrane transport function and immune system function were the lowest in the J 48 h group (down to 0.71-fold, *P* < 0.01) and J 6 h group (down to 0.79-fold, *P* < 0.05), respectively. Conversely, the relative abundance levels of peroxisome function and PPAR signaling pathway function were highest in the J 48 h group (up to 1.78-fold, *P* < 0.01) and J 96 h group (up to 1.65-fold, *P* < 0.01), respectively.

### 3.8. CCA analysis of intestinal microbial community, Pb content, and intestinal physicochemical factors

**Figure 8:**
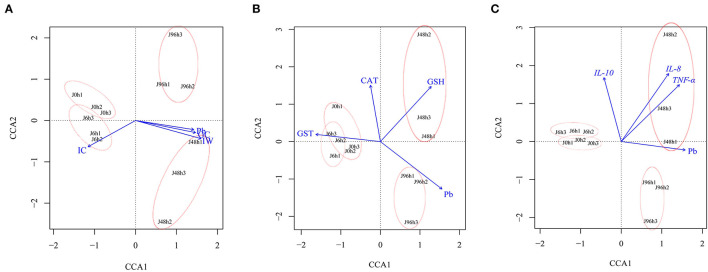
Canonical correlation analysis (CCA) between intestinal microbial community structure of crucian carp and **(A)** intestinal structure (IC, Intestine crypt relative depth; GC, Goblet cells relative number; IW, Intestinal wall relative thickness), **(B)** antioxidant factor (*CAT, GST, GSH*), **(C)** immune factors (*IL-8, IL-10, TNF-*α), and Pb content.

CCA analysis showed that the microbial community was closely related to intestinal Pb content, antioxidant factors (*CAT, GST*, and *GSH*), and immune factors (*IL-10, IL-8*, and *TNF- a*; Figure 8). After Pb exposure, the intestinal microbiota structure in the J 0 h and J 6 h groups showed high similarity and short distance between each other. The changes in the intestinal microbial community in the J 48 h and J 96 h groups were particular, which with large distances from the J 0 h group. Among them, the intestinal microbial changes in the J 48 h group were positively correlated with goblet cell number, intestinal wall, *GSH, IL-8*, and *TNF-a*, while the intestinal microbial structure in the J 96 h group was positively correlated with Pb content.

## 4. Discussion

Pb is widely present in the atmosphere, soil, and water and is a major pollutant in environmental monitoring and pollution control, with high toxicity to environmental organisms (Malik and Ahmad, 2002; Cruz et al., 2006; Melanie et al., 2015; Behera et al., 2021; Rout et al., 2022b). It can change the body function by breaking the intestinal wall barrier, destroying the intestinal structure, and changing the expression levels of some important genes in fish (Xia et al., 2018; Shi et al., 2020). Therefore, it is necessary to establish an effective toxicological evaluation of Pb. In this study, the effects of Pb on the morphological structure, antioxidant capacity, and immune capacity of the intestine of crucian carp under the SC were analyzed. It was found that Pb was enriched in the intestine of crucian carp and changed the morphological structure, antioxidant factors, and immune factors of the intestine. To further understand the effect of Pb on the intestinal microbial community of crucian carp, we used high-throughput sequencing technology to detect the composition of the intestinal microbial community of crucian carp. We found that there were significant correlations between the changes in the intestinal microbial community and intestinal structure indicators, antioxidant factors, and immune factors, indicating that the fish intestinal microbiota can be used as an important reference index for water pollution evaluation.

Intestine is an important organ for fish digestion, metabolism, endocrine regulation, and immunity (Buddington et al., 1997). Due to its communication with the external water environment, the intestine is one of the first organs to be affected by environmental pollutants, which is also one of the main organs for pollutants to accumulate (Yin et al., 2020). Therefore, the state of the intestine is often used as a good indicator for evaluating water quality and is a suitable model for studying the effects of environmental pollution (Ted et al., 2003; Karami et al., 2012; Li et al., 2014). Previous studies have shown that Pb accumulation can induce oxidative stress in the intestine of aquatic organisms (Duan et al., 2021). *CAT, GST*, and *GSH* play important roles in protecting tissues from oxidative stress. In multiple research studies, it was found that *CAT* plays an important role in the oxidative stress pathway against heavy metals, *GST* is a key factor in protecting organisms from oxidative stress, and *GSH* is involved in metal detoxification in animals (Narayanan et al., 2018; Yin et al., 2020). Therefore, *CAT, GST*, and *GSH* are generally considered as biomarkers of oxidative stress induced by exogenous exposure to aquatic organisms (Elia et al., 2003; Yin et al., 2020). In this study, the expression levels of *CAT, GST*, and *GSH* genes in the intestinal tissue increased, which further indicated that oxidative stress occurred in the intestine after Pb exposure. In this study, Duan's research suggested that Pb exposure will cause the occurrence of intestinal inflammation in fish, which was confirmed by the mRNA expression changes in immune-related genes *(TNF-*α, *IL-1*β, *IL-8*, and *IL-10*) (Duan et al., 2021). The occurrence of inflammation will lead to histopathological changes in the fish gut, such as enlarged intestinal wall, increased goblet cell numbers, and leukocyte infiltration (Liu et al., 2020). In this research, we observed intestinal histopathological changes in crucian carp, including enlargement of the intestinal wall, reduction of intestinal crypt length, increase in the number of goblet cells, and infiltration of leukocytes, which further promote the deepening of inflammation (Lofgren et al., 2002; Qiao et al., 2019; Liu et al., 2020).

Intestinal microbiota is an intricate and unique microbial community in the body, which is closely related to the body's metabolism, immunity, and behavior (Premysl et al., 2011; Wang et al., 2019; Zhang et al., 2020). Decreased microbial diversity and altered composition are considered to be the indicators for evaluating the occurrence of intestinal diseases (Xiong et al., 2015; Liu et al., 2020). This study found that Pb exposure reduced the intestinal microbiota diversity of crucian carp. Decreased microbial diversity may cause disorders such as intestinal microbial community and immune function, causing oxidative stress and leading to the occurrence of diseases (Goyette et al., 2007; Ganz et al., 2011; Kuno et al., 2016; Passos and Moraes, 2017). Therefore, the pathological response of lead exposure in the intestine of crucian carp may be related to the decrease in microbial diversity.

Waterborne lead significantly alters intestinal microbiota composition and abundance of fish. At the phylum level, *Proteobacteria* and *Bacteroidetes* became the most abundant bacteria in the intestine after Pb exposure implying that the stability of intestinal microbiota was influenced by Pb, which might affect the immune function, antioxidant function, and induce inflammation of crucian carp (Supplementary Figure S4). Feris and Kasemodel's research studies reported that gram-negative bacteria *Proteobacteria* are resistant to heavy metals, so there is no significant change in Pb-polluted areas (Feris et al., 2003; Kasemodel et al., 2019). Combined with previous research, *Proteobacteria* have a better heavy metal tolerance, so in the early stage, it becomes the dominant bacteria in the intestine after Pb stress (Hall and Meyer, 1998; Ancion et al., 2010). *Bacteroidetes*, the abundance of which increased significantly in the later stages of Pb exposure, are closely related to the induction of intestinal diseases (Zitomersky et al., 2013). Meanwhile, the increased abundance of *Bacteroidetes* can promote the occurrence of intestinal inflammation, which was further confirmed by the expression levels of immune genes in this study (Marchesi et al., 2016). Interestingly, intestinal microbiota network analysis found that the most abundant bacteria, OTU1 (belonging to *Bacteroidetes*), were positively correlated with OTU10 and OTU27 (belonging to *Proteobacteria*; Figure 6). These results imply that the increase in the abundance of *Proteobacteria* in the early stage may promote the increase in the abundance of *Bacteroides* in the later stage, after Pb exposure. On the contrary, *Firmicutes*, the dominant gram-positive bacteria present in the intestine of crucian carp, were significantly decreased after Pb exposure. Bacteria exhibited differential responses to Pb exposure, possibly because the cell membranes of gram-positive bacteria had highly electronegative chemicals that could bind heavy metal ions and destroy the function of bacterial membranes (Hyyryläinen et al., 2000; Huang et al., 2002; Lemire et al., 2013).

The family Vibrionaceae, belonging to the phylum Proteobacteria, which is a major pathogen that can cause severe economic loss in fish farming, was significantly increased in abundance under the influence of Pb (Sonia et al., 2015)., belonging to the phylum Bacteroidetes, which has been reported as a main fish pathogen in multiple salmon farms, widely recognized as one of the main sources of disease and economic loss of aquaculture (Nicolas et al., 2008). More than that, members of Bacteroides and Weeksellaceae have been found to promote inflammation and disrupt the formation of intestinal villi in fish (Liu et al., 2020). In this study, the changes in intestinal morphology may be related to the upregulation of the abundance of Weeksellaceae. On the contrary, Erysipelotrichaceae, which belongs to the phylum Tenericutes, had decreased after Pb exposure. Previous studies have found that the decline in the expression of Erysipelotrichaceae is significantly related to intestinal irritable bowel syndrome (Yang et al., 2016). Hence, the increase in the expression of Weeksellaceae, Vibrionaceae, and Flavobacteriaceae and the decrease in the expression of Erysipelotrichaceae might provide opportunities for the disease in the intestine of fish (Figure 4).

After Pb exposure, the deviated abundance value of the genus *Aeromonas* was reduced (Figure 5). Previous studies suggested that *Aeromonas* was resistant to heavy metals, such as Cd, Cu, and Cr (Kim et al., 2014). However, the deviation value of *Aeromonas*, in this study, was reduced, possibly because *Aeromonas* did not have Pb resistance. Conversely, the dominant levels of *Sediminibacterium* and *Chryseobacterium* increased significantly after Pb exposure. *Sediminibacterium* belongs to *Bacteroidetes*, and *Chryseobacterium* belongs to *Flavobacteria*, both are common pathogenic bacteria that cause inflammation in the intestines of fish and lead to fish death (Qu and Yuan, 2008; Satcolu et al., 2021). In addition, with the accumulation of Pb exposure time, the IBR index of the major bacteria at the genus level of the intestine of crucian carp increased, which reflects a gradual increase in the impact on the main bacteria in the intestine with the accumulation of Pb, further proved that the intestinal microbial community disorder of crucian carp caused by Pb was difficult to recover (Sanchez et al., 2013).

It was found that alterations in the intestinal microbiota would affect the intestinal structure and function of fish (Geovanny and Jose, 2010; Navarrete et al., 2013; Gao et al., 2017). As expected, the results of canonical correlation analysis indicated that changes in intestinal structure indices, as well as increased expression of immune factors in fish, were closely related to the changes in intestinal microbial communities (Figure 8). Surprisingly, this study also found that there is a correlation between antioxidant factors and intestinal microbiota, possibly because the imbalance of the intestinal microenvironment can promote intestinal oxidative stress. Crucian carp is an omnivorous fish and contains microorganisms in its diet (Li et al., 2013). Previous studies have also found that microorganisms can improve the physiological functions of crucian carp (Li Y. N. et al., 2015). Thus, the changes in the structure and function of the intestine may be closely related to intestinal microorganisms. The results of our study indicated that changes in intestinal microbiota will cause oxidative stress and affect the structure and immune function of the intestine. This was also confirmed by the functional changes (membrane transport, immune system, and peroxisome) of PICRUST analysis (Figure 7). In summary, when Pb pollution in the water environment causes structural changes and imbalances in the intestinal microbiota of crucian carp, the structure and function of the crucian carp will be affected.

## 5. Conclusion

In this study, we report the effects of Pb pollution on the intestine of crucian carp based on bioaccumulation, histopathology, gene expression, and high-throughput analysis for a comprehensive toxicological assessment of lead pollution in the aquatic environment. The results of this study indicate that waterborne Pb exposure cause structural damage, oxidative stress, and immune responses in the intestine of crucian carp. It is worth mentioning that after lead exposure, the changes in the abovementioned intestinal indicators may be closely related to intestinal microbiota. We found that the intestinal microbiota are sensitive to Pb pollution and altered the composition of the microbiota, increasing the abundance of pathogenic bacteria at an early stage, leading to intestinal disorders, and further aggravating the effect of Pb on the structure and function of the damaged intestine. In conclusion, our study provides the first report of comprehensively toxicological effects of Pb pollution on crucian carp and finds the relationship between the gut microbiota, morphology, antioxidant factors, and immune factors of crucian carp. The results of this study provide a basis for the link between gut microbiome changes and heavy metal toxicity, and gut microbiota can be used as biomarkers for the evaluation of heavy metal pollution.

## Data availability statement

The datasets presented in this study can be found in online repositories. The names of the repository/repositories and accession number(s) can be found below: NCBI- PRJNA984177.

## Ethics statement

All animal experimental procedures were carried out in accordance with the Regulations for Animal Experimentation of South China Normal University (SCNU-SLS-2020-001), and the animal facility was based on the National Institutes of Health guide for the care and use of Laboratory Animals (NIH Publications No. 8023). The study was conducted in accordance with the local legislation and institutional requirements.

## Author contributions

HL: writing—reviewing and editing, investigation, and visualization. HZ: investigation and writing—reviewing and editing. QY, SZ, and FZ: writing—reviewing and editing and resources. XT: writing—reviewing and editing. SF: conceptualization, writing—reviewing and editing, investigation, and resources. All authors contributed to the article and approved the submitted version.
