# Supplementary material for: Lead induced structural and functional damage and microbiota dysbiosis in the intestine of crucian carp (Carassius auratus)
Source: Front Microbiol. 2023 Sep 4;14:1239323. doi: 10.3389/fmicb.2023.1239323 (PMC10507410; doi:10.3389/fmicb.2023.1239323)
Supplement: Supplementary file 1 [file Image_1.pdf]

Supplementary Figure Legends

FIGURE S1. Comparison of OTUs in different groups by Venn diagram.

FIGURE S2. Rarefaction curve of different samples.

FIGURE S3. The effects of Pb on the richness and diversity of microbiota in the crucian carp's intestines in each sample. A. Ace index. B. Chao1 index. C. Shannon index. D. Simpson index.

FIGURE S4. The intestinal microbiota composition of the crucian carp's intestines at phylum level.

FIGURE S1

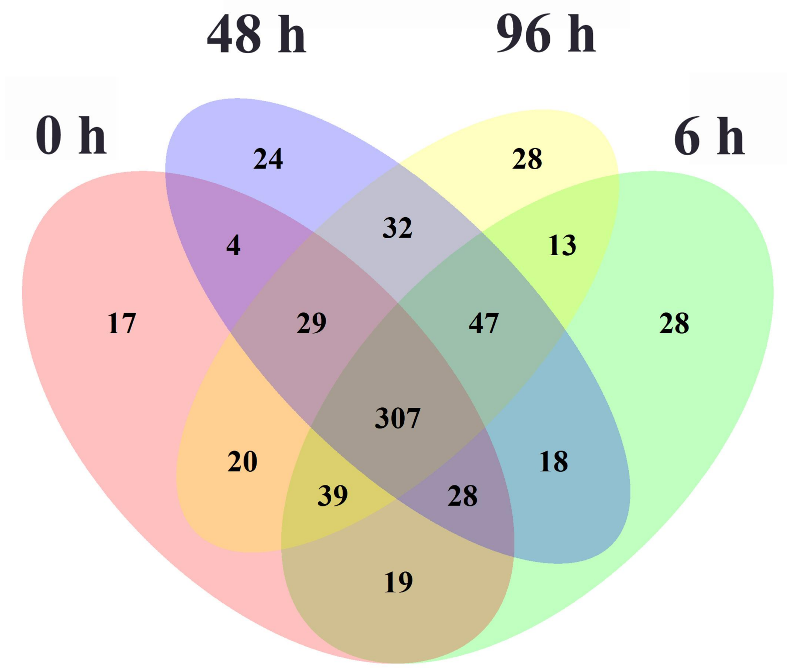

FIGURES2

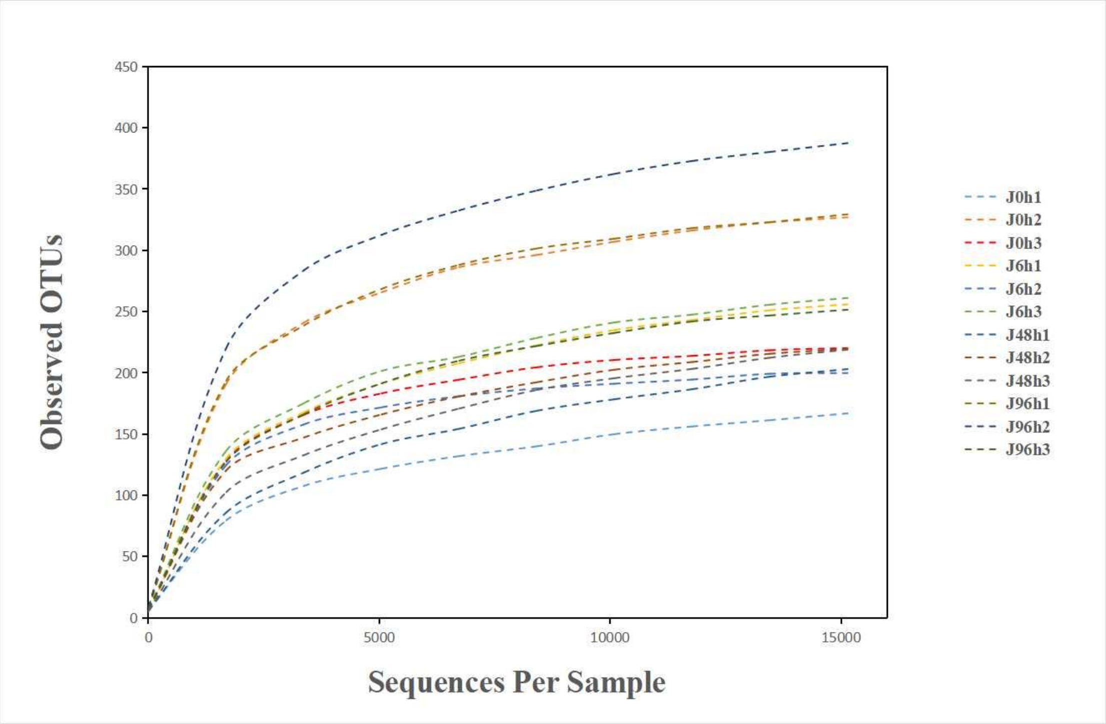
Observed OTUs
Sequences Per Sample
450
400
350
300
250
200
150
100
50
0
0
5000
10000
15000
J0h1
J0h2
J0h3
J6h1
J6h2
J6h3
J48h1
J48h2
J48h3
J96h1
J96h2
J96h3

FIGURE S3

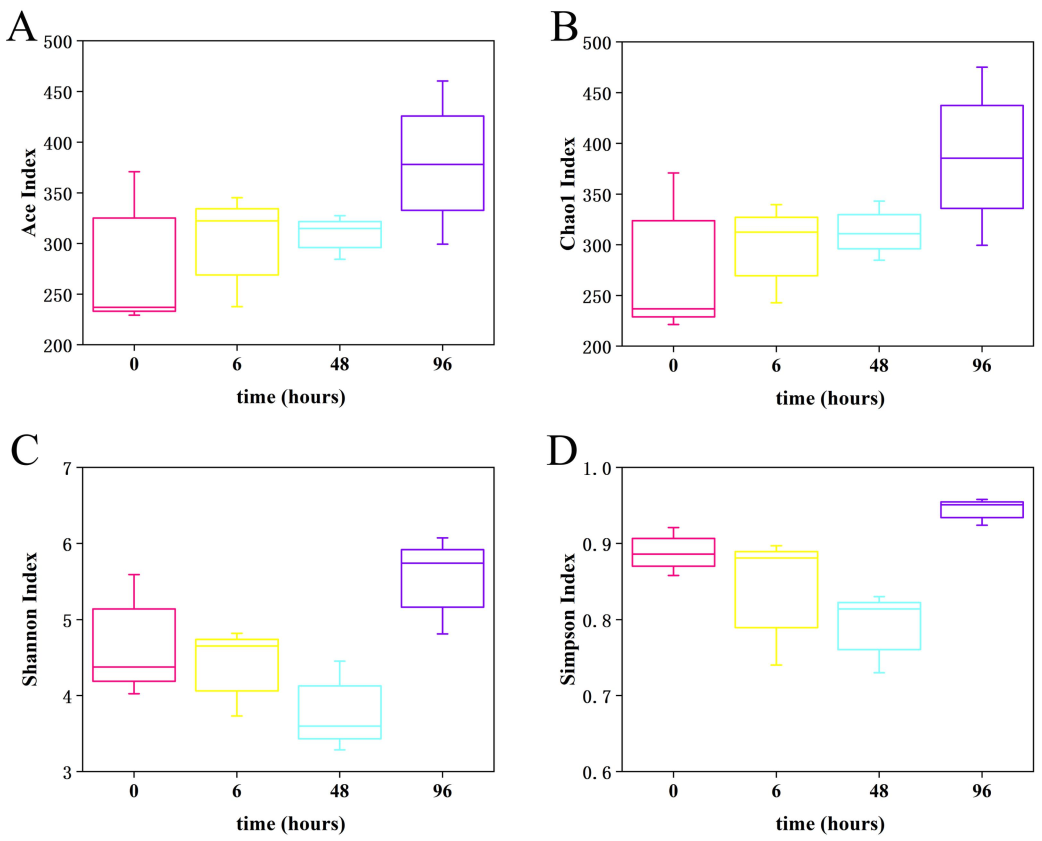
A
B
C
D
Ace Index
Chao1 Index
Shannon Index
Simpson Index
500
450
400
350
300
250
200
7
6
5
4
3
1.0
0.9
0.8
0.7
0.6
0
6
48
96
time (hours)

FIGURE S4

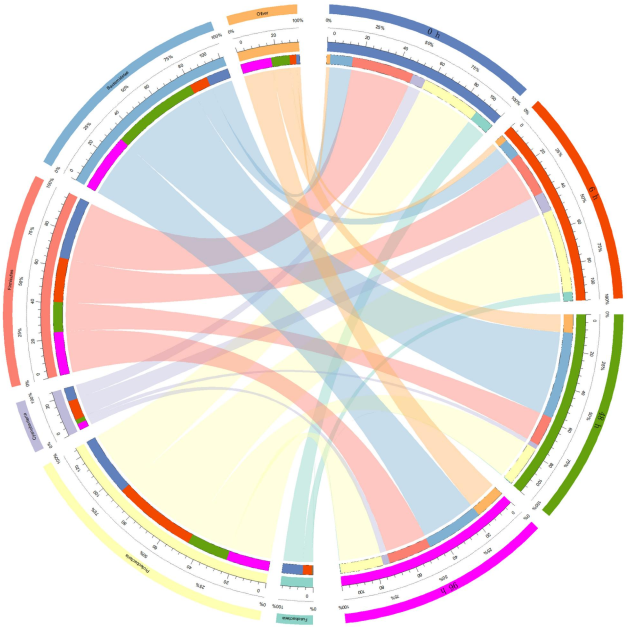
0 h
6 h
48 h
96 h
Other
Bacteroidetes
Firmicutes
Cyanobacteria
Proteobacteria
Fusobacteria
